# Supplementary material for: Identification of essential oils with activity against stationary phase Staphylococcus aureus
Source: BMC Complement Med Ther. 2020 Mar 24;20:99. doi: 10.1186/s12906-020-02898-4 (PMC7092464; doi:10.1186/s12906-020-02898-4)
Supplement: Supplementary file 1 — Additional file 1: Table S1. Chemical compositions of the most active essential oils against S. aureus [file 12906_2020_2898_MOESM1_ESM.docx]

**Table S1. Chemical compositions of the most active essential oils against *S. aureus***

| **Essential oil** | **Plant** | **Part** | **Components** | **Content (v/v)** | **Reference** |
| --- | --- | --- | --- | --- | --- |
| Oregano | *Origanum vulgare* | herbs | Carvacrol | 80.4% | [1] |
|  |  |  | Thymol | 6.6% |  |
|  |  |  | p-Cymene | 4.3% |  |
|  |  |  | 1,8-Clneole | 1.4% |  |
|  |  |  | Linaiool | 1.3% |  |
| Cinnamon bark | *Cinnamomum zeylanicum* | bark | Cinnamaldehyde | 71.8% | [2] |
|  |  |  | Eugenol | 5.1% |  |
|  |  |  | α-Pinene | 4.6% |  |
|  |  |  | Linalool | 3.5% |  |
|  |  |  | Cinnamyl acetate | 3.4% |  |
| Allspice | *Pimenta officicalis* | berries | Eugenol | 82% | [3] |
|  |  |  | β-Caryophyllene | 6% |  |
|  |  |  | Methyleugenol | 5% |  |
|  |  |  | α-Humulene | 1% |  |
|  |  |  | α-Selinene | 0.47% |  |
| Thyme white | *Thymus zygis* | leaves | Thymol | 46.2% | [4] |
|  |  |  | p-terpinene | 19.7% |  |
|  |  |  | Carvacrol | 9.4% |  |
|  |  |  | Linalool | 4.0% |  |
|  |  |  | β-pinene | 3.9% |  |
| Lemongrass | *Cymbopogon citratus* | herbs | Geranial | 39.7% | [5] |
|  |  |  | Neral | 30.1% |  |
|  |  |  | Geraniol | 8.4% |  |
|  |  |  | Geranyl acetate | 4.9% |  |
|  |  |  | β-Caryophyllene | 1.5% |  |
| Palmarosa | *Cymbopogon martinii* | grass | Geraniol | 79% | [6] |
|  |  |  | Geranyl acetate | 9.0% |  |
|  |  |  | Linalool | 2.6% |  |
|  |  |  | β-Caryophyllene | 2% |  |
|  |  |  | Trans-β-ocimene | 1.5% |  |
| Amyris | *Amycris balsamifera* | wood | Valerianol | 25.0% | [7] |
|  |  |  | α-Elemol | 10.0% |  |
|  |  |  | α-Eudesmol | 9.4% |  |
|  |  |  | α-Eudesmol | 8.2% |  |
|  |  |  | γ-Eudesmol | 7.7% |  |
| Sandalwood oil | *Santalum spicatum* | wood | (Z)-Santalol | 27.3% | [8] |
|  |  |  | (Z)- β-Santalol | 10.6% |  |
|  |  |  | (Z)-Nuciferol | 10.2% |  |
|  |  |  | α-Bisabolol | 8.7% |  |
|  |  |  | cis-Farnesol | 6.3% |  |

References

1. Compositions of essential oils Available online: https://www.planttherapy.com/test_reports/Oregano%20organic%20O50100.pdf (accessed on 24 December 2019).

2. Feng J, Zhang S, Shi W, Zubcevik N, Miklossy J, Zhang Y. Selective Essential Oils from Spice or Culinary Herbs Have High Activity against Stationary Phase and Biofilm Borrelia burgdorferi. Front Med (Lausanne). 2017;4:169. Epub 2017/10/28. doi: 10.3389/fmed.2017.00169. PubMed PMID: 29075628; PubMed Central PMCID: PMCPMC5641543.

3. Compositions of essential oils Available online: https://www.theplantguru.com/amfile/file/download/file_id/1/product_id/622/ (accessed on 8 November 2019)

4. Compositions of essential oils Available online: https://www.planttherapy.com/thyme-thymol-essential-oil?v=333 (accessed on 24 December 2019).

5. Compositions of essential oils Available online: https://www.planttherapy.com/test_reports/Lemongrass%20Organic%20L90102.pdf (accessed on 24 December 2019).

6. Compositions of essential oils Available online: https://www.planttherapy.com/test_reports/Palmarosa%20Organic%20PK0100.pdf (accessed on 24 December 2019).

7. Compositions of essential oils Available online: https://www.planttherapy.com/test_reports/Amyris%20AHO100.pdf (accessed on 24 December 2019).

8. Compositions of essential oils Available online: https://www.planttherapy.com/test_reports/Sandalwood%20S20101.pdf (accessed on 24 December 2019).
